# Proteomic Phosphosite Analysis Identified Crucial NPM-ALK-Mediated NIPA Serine and Threonine Residues

**DOI:** 10.3390/ijms20164060

**Published:** 2019-08-20

**Authors:** Anina Gengenbacher, Alina Müller-Rudorf, Teresa Poggio, Linda Gräßel, Veronica I. Dumit, Stefanie Kreutmair, Lena J. Lippert, Justus Duyster, Anna L. Illert

**Affiliations:** 1 Department of Hematology and Oncology, Freiburg University Medical Center, Albert-Ludwigs-University of Freiburg, 79106 Freiburg, Germany; 2 German Cancer Consortium (DKTK) and German Cancer Research Center (DKFZ), 69120 Heidelberg, Germany; 3 Center for Biological Systems Analysis (ZBSA), University of Freiburg, 79104 Freiburg, Germany

**Keywords:** ALCL, NPM-ALK, NIPA (ZC3HC1), phosphoproteomics

## Abstract

Anaplastic large-cell lymphoma (ALCL) is an aggressive non-Hodgkin lymphoma that shows in 60% of cases a translocation t(2;5)(p23;q35), which leads to the expression of the oncogenic kinase NPM-ALK. The nuclear interaction partner of ALK (NIPA) defines an E3-SCF ligase that contributes to the timing of mitotic entry. It has been shown that co-expression of NIPA and NPM-ALK results in constitutive NIPA phosphorylation. By mass spectrometry-based proteomics we identified nine serine/threonine residues to be significantly upregulated in NIPA upon NPM-ALK expression. Generation of phospho-deficient mutants of the respective phospho-residues specified five serine/threonine residues (Ser-338, Ser-344, Ser-370, Ser-381 and Thr-387) as key phosphorylation sites involved in NPM-ALK-directed phosphorylation of NIPA. Analysis of the biological impact of NIPA phosphorylation by NPM-ALK demonstrated that the ALK-induced phosphorylation does not change the SCF^NIPA^-complex formation but may influence the localization of NIPA and NPM-ALK. Biochemical analyses with phospho-deficient mutants elucidated the importance of NIPA phosphorylation by NPM-ALK for the interaction of the two proteins and proliferation potential of respective cells: Silencing of the five crucial NIPA serine/threonine residues led to a highly enhanced NIPA-NPM-ALK binding capacity as well as a slightly reduced proliferation in Ba/F3 cells.

## 1. Introduction

Anaplastic large cell lymphoma (ALCL) belongs to the group of aggressive non-Hodgkin’s lymphomas (NHL) and was first described as a distinct tumor entity in 1985 [1]. It is characterized by the expression of the CD30 receptor (Ki-1), an anaplastic morphology and a null-cell or T-cell phenotype [2]. About 70–80% of ALK + ALCLs show the translocation t(2; 5)(p23; q35), thereby expressing the chimeric NPM-ALK fusion protein [3]. The 80 kDa fusion protein NPM-ALK was first described in 1994. The fusion of the N-terminal portion of the *npm* gene (chromosome 5) with the C-terminal portion of the *alk* gene (chromosome 2) results in constitutive activation of the tyrosine kinase induced by NPM domain oligomerization and autophosphorylation of the ALK domain [4]. The consequence is a consecutive tumorigenesis by increased proliferation on the one hand and by inhibition of apoptosis on the other hand [5,6]. NPM-ALK stimulates numerous downstream targets and signaling pathways possibly involved in mediating its oncogenicity, including MEK/ERK, mTor, Pl3K/AKT and STAT3/5 [5,7,8,9]. Despite previous studies, the molecular mechanisms contributing to the distinct oncogenic features of NPM-ALK are still not fully understood.

In 2003, NIPA (nuclear interaction partner of ALK) was first identified as a protein of widespread expression and exclusive nuclear subcellular location in a “yeast two-hybrid screen” to identify phosphotyrosine-dependent interaction partners of the activated anaplastic lymphoma kinase (ALK) receptor tyrosine kinase [10]. NIPA was characterized as a human F-box protein that defines an SCF-type ubiquitin E3-ligase (SCF^NIPA^), which targets nuclear cyclin B1 for degradation in order to contribute to the timing of mitotic entry. The SCF^NIPA^-complex activity is regulated by cell-cycle-dependent phosphorylation of NIPA [11]. Recently, it has been shown that co-expression of NIPA with the oncogenic tyrosine kinase NPM-ALK also results in constitutive phosphorylation of NIPA [12]. In the present study, we aimed to identify the crucial sites involved in NIPA phosphorylation upon NPM-ALK expression as well as the functional impact of NPM-ALK-induced NIPA phosphorylation.

## 2. Results

### 2.1. Phosphorylation of NIPA in ALCL Cells Is Induced in an NPM-ALK-Dependent Manner

To demonstrate that NIPA is specifically phosphorylated by ALK expression and not by commonly upregulated pathways (i.e., MEK-pathway) in ALCL cells, we analyzed the phosphorylation of NIPA in ALK^+^ (DEL, TS, JB6) and ALK^-^ (Jurkat, FEPD, Mac-1) ALCL cell lines with regard to the shift of NIPA in electrophoretic mobility.

**Figure 1:**
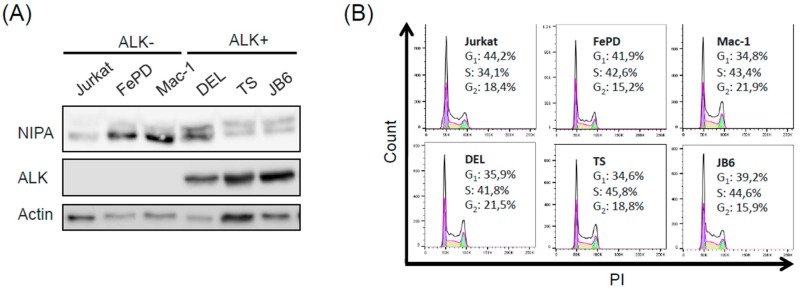
Phosphorylation of NIPA in anaplastic large-cell lymphoma (ALCL) cells is induced in an NPM-ALK-dependent manner (**A**) ALK^-^ and ALK^+^ cells were used to examine the phosphorylation status of NIPA with immunoblot analysis. (**B**) Cell staining with propidium iodide revealed equal amount of cells in G_2_/M to exclude cell-cycle-dependent phosphorylation differences.

The phosphorylation of NIPA is preferentially shown in ALK^+^ ALCL cell lines (Figure 1A), suggesting ALK dependent phosphorylation of NIPA. To rule out that different levels of phosphorylation are caused by ERK2-mediated phosphorylation during the interphase, we performed cell cycle analysis using propidium iodide (PI) staining with the same cells. As shown in the 2-dimensional cell cycle analyses, both groups presented an equal amount of cells in the G_2_/M-phase (Figure 1B), indicating that NIPA phosphorylation is not caused by an altered cell cycle distribution between the ALK^+^ and ALK^-^ ALCL cell entities. Therefore, we concluded that phosphorylation of NIPA seen by the electrophoretic mobility of the protein is correlated with NPM-ALK expression.

### 2.2. Proteomic Phosphosite Analysis Identified Five Crucial NPM-ALK-Induced NIPA Phosphosites

In order to identify the relevant phosphorylation sites of NPM-ALK-induced NIPA phosphorylation, we performed mass spectrometry (MS)-based proteomic phospho-screens (*n* = 5). For this purpose, Hek293T cells were transfected with FLAG-tagged NIPA and NPM-ALK vectors, harvested 24 h after transfection and prepared for mass spectrometry by immunoprecipitation (IP) of NIPA.

**Figure 2:**
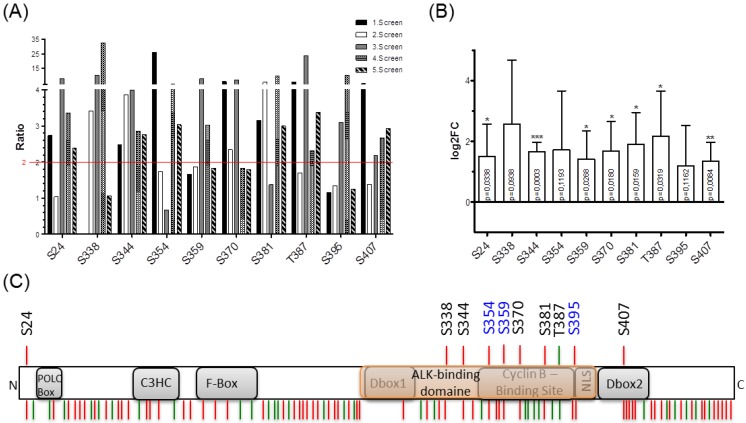
Mass spectrometry-based proteomic phosphosite analysis identified specific NPM-ALK induced NIPA phosphosites. (**A**) Overexpressed FLAG-tagged NIPA in 293T cells was immunoprecipitated and used for proteomic phosphosite analysis: Serine residues S24, S338, S344, S354, S370, S381, S387 and S407 appeared significantly upregulated upon NPM-ALK expression with a ratio >2 in more than 3 independent experiments. (**B**) Log2-fold change revealed S344 (*p* = 0.0003) to be highly significantly regulated, S407 (*p* = 0.0084) significant and S24 (*p* = 0.0338), S359 (*p* = 0.0268), S370 (*p* = 0.018), S381 (*p* = 0.0159) and T387 (*p* = 0.0319) as significant residues with induced phosphorylation status upon NPM-ALK expression. (**C**) Schematic overview of the phosphorylation sites within the human NIPA protein. Red: Serine; green: Threonine; NLS: Nuclear localisation signal; Dbox: Destruction box; C3HC: Zink finger binding motif; F-Box: F-Box domain; C: C-terminus; N: N-terminus; blue: Previously identified amino acids phosphorylated at G_2_/M; black: Possible amino acids phosphorylated by NPM-ALK identified with proteomic phosphosite analysis (* *p* < 0.05, ** *p* < 0.01, and *** *p* < 0.001).

To identify crucial amino acids (AA) in NIPA, which were specifically phosphorylated upon NPM-ALK expression, we followed a proteomics approach. In this way, we detected changes higher than 2.0 in the ratio of identified phosphosites in NPM-ALK expression relative to NPM-ALK negative cells. Thereby, we could detect eight specific AA which were significantly upregulated in more than three independent experiments (Figure 2A). Analysis of the Log2-fold change revealed six serines and one threonine as significantly phosphorylated in NPM-ALK expressing versus non-expressing cells (Figure 2B). Combination of these statistical methods finally resulted in six AA of NIPA significantly phosphorylated in NPM-ALK expressing HEK293T cells. Interestingly, we found the blue-labeled amino acids (S354, S359, S395), previously described to be phosphorylated cell-cycle dependent [12,13], to be partly altered, but not significantly induced in NPM-ALK expressing cells, therefore suggesting an NPM-ALK dependent phosphorylation of NIPA exceeding the cell-cycle-dependent regulation of the protein.

To further precisely define the essential phosphorylation sites, we generated different truncated NIPA mutants expressing only AA1-402 or lacking AA310-402. Co-expression of these FLAG-tagged mutants with NPM-ALK in Hek293T cells demonstrated abolished NIPA phosphorylation in the mutant lacking AA310-402, whereas the wildtype as well as the NIPA mutant expression AA1-402 showed distinct NIPA phosphorylation upon NPM-ALK expression seen by the shift in electrophoretic mobility of the protein. Therefore, we can biochemically prove that the phosphorylation sites between AA310-402 (S338; S344; S370; S381 and S387) are most likely crucial for NIPA phosphorylation upon NPM-ALK expression.

**Figure 3:**
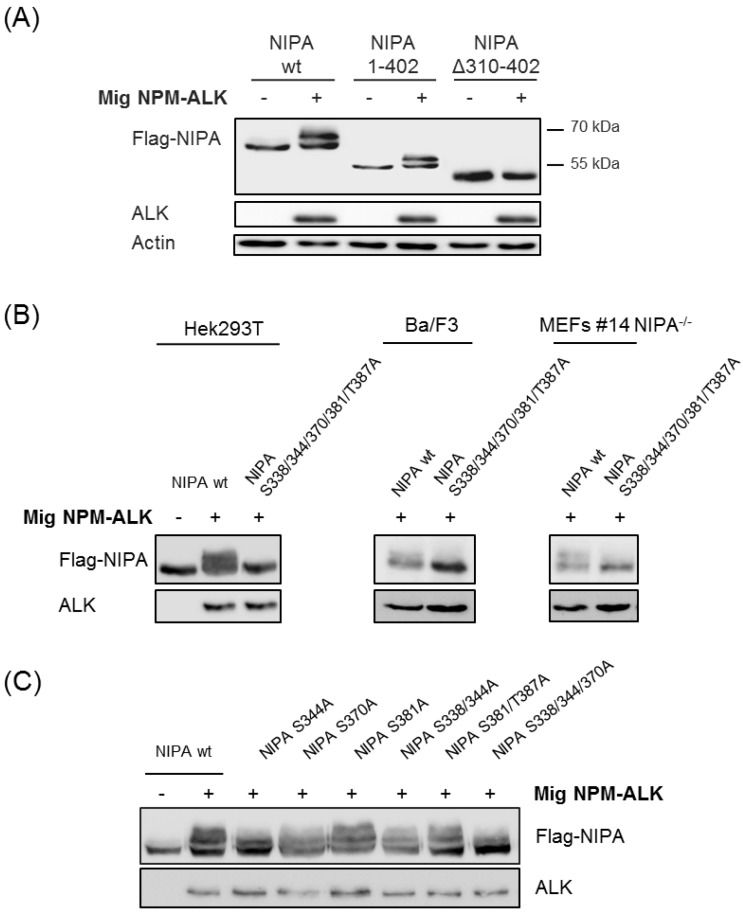
NPM-ALK induces phosphorylation of NIPA at Ser-338, Ser-344, Ser-370, Ser-381 and Thr-381. (**A**) Hek293T cells were transiently transfected with pCDNA Flag hNIPA wt, pCDNA Flag hNIPA 1-402, pCDNA Flag hNIPA Δ310-402 +/− Mig NPM-ALK and subjected to western blot. Immunoblotting confirmed that region 310–402 seems to be crucial for NPM-ALK mediated NIPA phosphorylation. (**B**,**C**) Hek293T cells were transiently transfected with pCDNA Flag hNIPA wt, pCDNA Flag hNIPA S344A, pCDNA Flag hNIPA S370A, pCDNA Flag hNIPA S381A, pCDNA Flag hNIPA S338/344A, pCDNA Flag hNIPA S381/387A, pCDNA Flag hNIPA S338/344/370A and pCDNA Flag hNIPA S338/344/370/381/387A +/− Mig NPM-ALK. Ba/F3 cells and *Nipa* −/− MEFs were stably transfected with pMSCV PIC Flag hNIPA wt and pMSCV PIC Flag hNIPA S338/344/370/381/387A +/− Mig NPM-ALK. Immunoblotting showed impaired phosphorylation for the phospho-deficient NIPA mutant S338/344/370/381/387A.

To further specify the crucial phosphosites, we created phosphodeficient NIPA mutants by replacing the respective serine or threonine residue with an alanine and introduced these constructs in HEK293T, Ba/F3 and *Nipa* deficient mouse embryonic fibroblasts (MEFs). Co-expression of the quintuplicate NIPA phosphodeficient mutant (S338/344/370/381/387A) abolished the phosphorylation capacity of NPM-ALK to NIPA as seen by the unphosphorylated, unshifted mutant form of NIPA in HEK293T, Ba/F3 and *Nipa* deficient MEFs (Figure 3B, Supplemental Figure S1). However, co-expression of the different single, double and triple NIPA phospho-deficient mutants indicate no differences in NIPA phosphorylation of NPM-ALK by phospho-deficient mutations of S381A and S381/387A, but exhibit reduced phosphorylation ability of the single S344A and S370A NIPA upon NPM-ALK expression (Figure 3C). Immunoblot analyses of the triple mutation S338/344/370A seems to abrogate NIPA phosphorylation by NPM-ALK similarly to the quintuplicate NIPA phospho-deficient mutant. As minimal residual phosphorylation of the triple phospho-deficient mutant NIPA S338/344/370A cannot be completely excluded by biochemical methods, we propose that five residues (serine 338, 344, 370, 381 and threonine 387) are essential and crucial for NPM-ALK-induced NIPA phosphorylation.

The tyrosine kinase NPM-ALK itself is not able to phosphorylate serine/threonine residues of NIPA, and data exist that MAP-Kinases like ERK1- and -2 are important for the NPM-ALK-induced NIPA phosphorylation [12]. However, NIPA harbors a Polo-box motif at its N-terminus, thereby providing a possible interaction site for the serine/threonine kinase Polo-like kinase 1 (PLK1). Indeed, we were able to co-immunoprecipitate NIPA and PLK1 in the ALK-positive cell lines Karpas and SUDHL-1 (Supplemental Figure S2A). However, when treating HEK293T cells expressing both NIPA and NPM-ALK with the PLK1 inhibitor Volasertib we could not detect any effect on NIPA phosphorylation (Supplemental Figure S2B). Thus, we concluded that PLK1 is most likely not the kinase responsible for the NPM-ALK-induced phosphorylation. Interestingly, consensus sequence analyses of the identified phosphosites predicted the MAP kinases ERK1/2, JNK1 and p38MAPK (Supplemental Figure S2C), thereby again providing evidence for an involvement of MAPK in the phosphorylation of NIPA.

### 2.3. NPM-ALK-Mediated NIPA Phosphorylation Does Not Change the SCF^NIPA^-Complex Formation but Might Influence the NIPA Localization at the Nucleus

**Figure 4:**
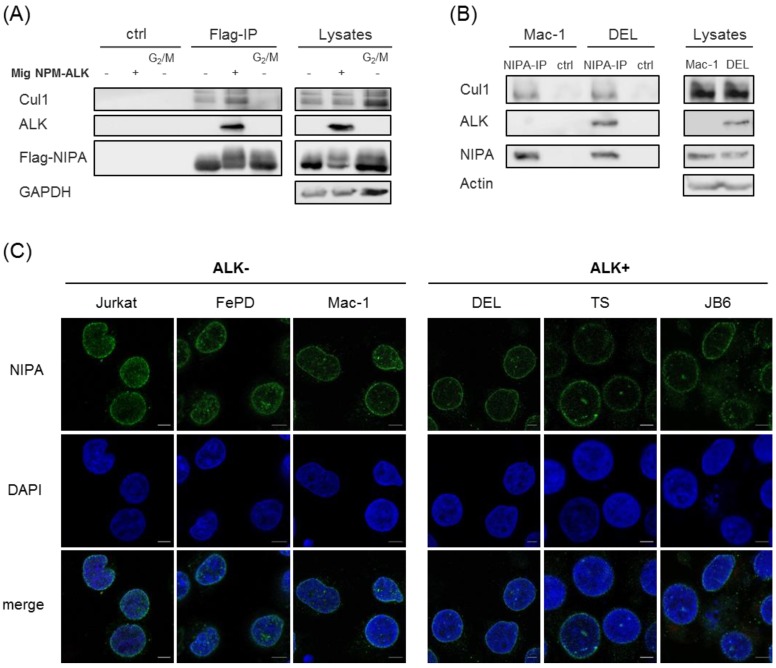
NPM-ALK-mediated NIPA phosphorylation does not change the SCF^NIPA^-complex formation but might influence the NIPA localization at the nucleus. (**A**) Hek293T cells were transiently transfected with pCDNA Flag hNIPA wt +/− Mig NPM-ALK and used for Flag-IP. (**B**) ALK^−^ ALCL cells (Mac-1) and ALK^+^ ALCL cells (DEL) confirmed binding of the SCF-core-complex binding to endogenous NIPA. (**C**) ALK^+^ and ALK^−^ cells were used for immunofluorescence and displayed slight differences in endogenous NIPA localisation with a shift to the nuclear membrane in ALK^+^ cells. NIPA was stained with Alexa Fluor 488 (green). DAPI (blue) was used to stain the nucleus (Scale bars: 5 µm).

NIPA has already been characterized to be involved in the regulation of the cell cycle [11]. As E3 ubiquitin ligase, it is part of an SCF^NIPA^-complex that targets cyclin B1 in interphase. At G_2_/M transition NIPA is phosphorylated by ERK2, leading to the dissociation from the SCF-complex and thereby its inactivation [12,13]. In order to investigate whether NPM-ALK-induced phosphorylation of NIPA may also lead to dissociation of the protein from the SCF core complex, we investigated the NIPA-SCF binding by co-immunoprecipitation. Flag-tagged NIPA and NPM-ALK were overexpressed in HEK293T cells, lysed and subjected to Flag-immunoprecipitation. As published, immunoblot analyses showed co-immunoprecipitation of NIPA and Cul1 as being part of the SCF complex in NPM-ALK negative cells [11,13]. Moreover, this co-immunoprecipitation could be abrogated in nocodazole treated G_2_/M lysates as previously shown, where phosphorylation of AA S354, 359 and 395 are involved [13]. Interestingly, phosphorylation of NIPA by NPM-ALK did not lead to dissociation of NIPA from the SCF core complex, as co-immunoprecipitation of NIPA with Cul1 could be clearly detected in NPM-ALK expressing cells. This again suggests that NPM-ALK-induced NIPA phosphorylation is independent of AA S354, 359 and 395 and does not affect the SCF^NIPA^ complex (Figure 4A). To confirm these results with endogenous proteins, we performed NIPA co-immunoprecipitation in ALK-positive (DEL) and ALK-negative (Mac-1) human ALCL cell lines. As shown in Figure 4B, Cul1 binding to NIPA could be detected to comparable levels in ALK^+^ and ALK^-^ cell lines, confirming correct SCF-NIPA formation despite NPM-ALK induced NIPA phosphorylation. We could show that NPM-ALK does not influence the assembly of the SCF^NIPA^-complex and therefore suggest that NPM-ALK-mediated phosphorylation of NIPA does not alter nuclear Cyclin B1 degradation.

It has already been shown that NIPA is strictly localized to the nucleus of the cell. As NPM-ALK is both nuclear and cytoplasmic [14], and shuttles between both cell compartments, we hypothesized that NIPA’s binding to NPM-ALK and its phosphorylation might alter the subcellular localization of the protein(s). Therefore, we used different ALK+ (DEL, TS, JB6) and ALK- (Jurkat, FEPD, Mac-1) ALCL cell lines and investigated endogenous NIPA localization by immunofluorescence staining after cytospin of the respective cells. As shown in Figure 4C, NIPA clearly localized the nucleus of ALK^+^ and ALK^−^ cells, with a slight localization shift of the protein from the nuclear center to the nuclear membrane in ALK-positive cells.

### 2.4. NIPA Phosphodeficiency Leads to Enhanced NPM-ALK Binding and Impaired Cell Proliferation in NPM-ALK-Positive Cells

As all identified NPM-ALK-mediated NIPA phosphorylation sites are localized to the ALK binding domain of NIPA (Figure 2C), we hypothesized that the NPM-ALK-induced phosphorylation of NIPA might alter the NIPA-NPM-ALK interaction. Therefore, we performed co-immunoprecipitation assays of NPM-ALK and different NIPA mutants.

**Figure 5:**
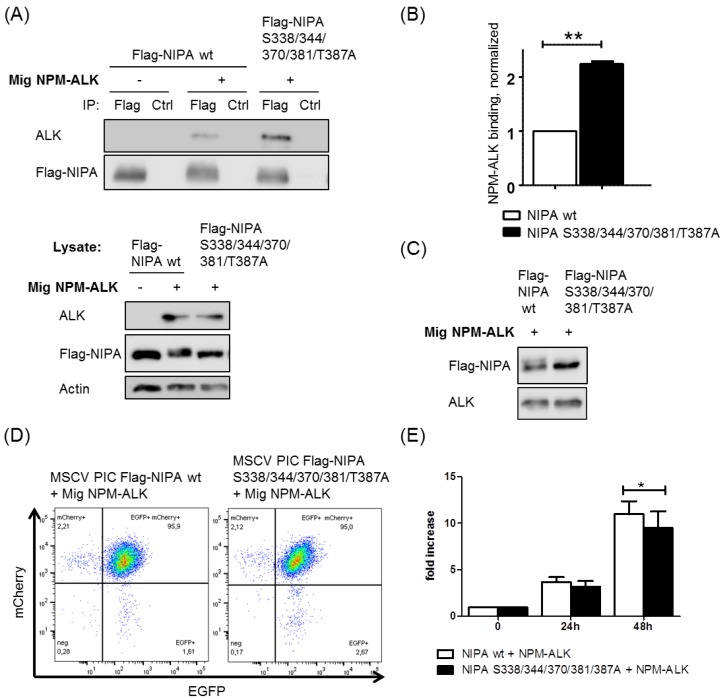
NIPA phospho-deficiency leads to enhanced NPM-ALK binding and impaired cell proliferation in NPM-ALK positive cells. (**A**) HEK293T cells were transiently transfected with pCDNA Flag hNIPA wt or pCDNA Flag hNIPA S338/344/370/381/387A +/− Mig NPM-ALK and used for Flag-IP. (**B**) Quantification of three independent experiments showed increased NPM-ALK binding ability to phospho-deficient NIPA S338/344/370/381/387A (** *p* < 0.01). (**C**) Ba/F3 cells were stably transfected with MSCV PIC Flag hNIPA wt or MSCV PIC Flag hNIPA S338/344/370/ 381/387A +/− Mig NPM-ALK. WB and (**D**) FACS analysis confirmed NIPA-mCherry and NPM-ALK-EGFP expression. (**E**) MTS assays were performed with 5000 cells and triplicates to evaluate the proliferative potential of the cells. Quantification of three independent MTS assays showed impaired growth in NPM-ALK^+^ BaF3 cells with NIPA S338/344/370/381/387A within 48 h (* *p* < 0.05).

Interestingly, we were able to show that the phospho-deficient NIPA S338/344/370/381/387A mutant binds NPM-ALK with a significantly higher affinity than wild-type NIPA (Figure 5A,B). Therefore, we conclude that the phosphorylation of NIPA upon NPM-ALK expression seems to limit the binding of NPM-ALK to NIPA, which we speculate could be important for the NPM-ALK activity and localization of NPM-ALK to the cytoplasm.

In order to further analyze the functional effects of NPM-ALK-mediated NIPA phosphorylation, we generated a Ba/F3 cell line expressing the NIPA wild type or the phospho-deficient NIPA S338/344/370/381/387A mutant, as well as NPM-ALK using retroviral infection. NIPA (mCherry) and NPM-ALK (EGFP) expression upon co-infection was confirmed by immunoblot and flow cytometry (Figure 5C,D). To study the influence of NPM-ALK-specific NIPA phosphorylation on cell proliferation, we performed an MTS proliferation assay. As shown in Figure 5E, the phospho-deficient NIPA S338/344/370/381/387A mutant showed a slight reduction in cell growth compared to wild type NIPA upon NPM-ALK expression. These results could be confirmed by soft agar proliferation assays with *Nipa*-deficient MEFs expressing NPM-ALK together with Flag-NIPA wt or Flag-NIPA S338/344/370/381/387A (Supplemental Figure S3A–C), but again, only a minor but significant effect could be observed. Therefore, we concluded that NPM-ALK-mediated NIPA phosphorylation seems to influence cell proliferation in an unfavorable manner, although these effects may not play a crucial role in NPM-ALK-mediated lymphomagenesis.

Taken together, we identified five serine/threonine residues (Ser-338, Ser-344, Ser-370, Ser-381 and Thr-387) as phosphorylation sites in NIPA to be significantly induced upon NPM-ALK expression with biological impact on NIPA-NPM-ALK interaction. Phosphorylation does neither change the SCF^NIPA^-complex formation nor influences the NIPA localization. Silencing of these NIPA serine/threonine residues led to a significantly increased NIPA-NPM-ALK binding capacity as well as slightly reduced proliferation in Ba/F3 cells and MEFs.

## 3. Discussion

The oncogenic tyrosine kinase NPM-ALK promotes tumorigenicity by binding and activating several proteins involved in antiapoptotic, promitogenic and transforming pathways. In addition, several transcriptional and cell cycle regulatory factors that play a role in NPM-ALK-mediated tumorigenesis have been recently identified [15]. However, the molecular mechanism contributing to the distinct oncogenic features of NPM-ALK is still not fully understood.

The F-box protein NIPA, which is involved in cell cycle regulation [11], has been identified as a binding partner of NPM-ALK leading to its constitutive phosphorylation [10,12]. In this study, we demonstrate cell-cycle-independent NIPA phosphorylation in NPM-ALK-positive ALCL cells. NPM-ALK-mediated NIPA phosphorylation did neither affect cell cycle rate nor SCF^NIPA^-complex structure, suggesting a cell-cycle-independent function of NIPA activated by this phosphorylation.

As oncogenic tyrosine kinase, NPM-ALK is known to phosphorylate and activate several proteins: STAT3 as a major player in ALCL is constitutively phosphorylated by binding to NPM-ALK, and thereby contributes to lymphoma pathogenesis [16,17,18,19,20]. Moreover, NPM-ALK recruits PI3K p85 subunit leading to the activation of the PI3K/Akt pathway contributing to ALK+ ALCL progression [21]. Additional proteins activated by NPM-ALK binding are, e.g., CD30 [22], PLC-γ [23], STAT1 [24] and the Src kinase pp60 [6], all being involved in tumorigenesis by enhancing cell proliferation.

Here, we identified five NPM-ALK-induced NIPA phosphorylation sites located on Ser-338, Ser-344, Ser-370, Ser-381 and Thr-387, which differ from the ERK2-induced serine residues phosphorylated during cell cycle progression [11,13]. Since the identified phosphorylation sites are serines and threonines it seems unlikely that the tyrosine kinase ALK phosphorylates NIPA directly. NPM-ALK stimulates numerous downstream targets and signaling pathways possibly involved in mediating its oncogenicity, thereby not only leading to tyrosine but also serine phosphorylation such as S9-GSK3β by PI3K/AKT [25]. Indeed, 506 phosphoproteins have been previously identified in NPM-ALK-expressing cells including a large number of serine/threonine phosphorylated proteins [26]. Numerous serine/threonine kinases activated by NPM-ALK (i.e., MEK/ERK, mTor, Pl3K/AKT) [5,7,8,9] could be considered as potential kinases for NIPA phosphorylation: The identified phosphorylation sites predominantly result in ERK1/2, JNK1 and p38MAPK recognition sites, which is in line with the publication of Illert et al. showing impaired NIPA phosphorylation upon MAPK inhibitor treatment in cells expressing NPM-ALK [12], thereby reinforcing the importance of the MAP-kinases for NIPA phosphorylation. Interestingly, the identified phosphorylation sites are located in a small area of the protein—which includes the ALK-binding domain [10]—suggesting a direct effect on the binding energy of the complex [27,28]. In fact, in this work the phospho-deficient NIPA mutant showed significantly increased NPM-ALK binding compared to wild type NIPA. NIPA has already been identified as a nuclear protein, while the subcellular localization of NPM-ALK is both nuclear and cytoplasmic [14]. It was already assumed that nuclear-located NIPA is able to relocalize ALK fusion proteins into the nucleus [10]. Therefore, we hypothesized that the interaction and phosphorylation of NIPA might influence the localization of NPM-ALK or NIPA. Here we could show that the phospho-deficient NIPA mutant binds NPM-ALK to a higher extent as phosphorylated NIPA and might thereby retain the protein in the nucleus. As only cytoplasmic NPM-ALK is catalytically active, whereas the nuclear portion is inactive because of heterodimerization with NPM1, the amount of cytoplasmic NPM-ALK is crucial for optimal lymphoma growth [14]. Thus, NPM-ALK might ensure its own cytoplasmic activity by NIPA phosphorylation. In line with these observations, we could show that phosphorylation of NIPA upon NPM-ALK expression slightly affects cell proliferation. NPM-ALK-positive cells expressing the phospho-deficient NIPA mutant showed significantly reduced cell growth compared to NIPA wild type cells. As NIPA phospho-deficiency changes the proliferation potential only to a minor extent, we concluded that these effects may not play a crucial role in NPM-ALK-mediated lymphomagenesis. However, these findings provide novel insights for further analyses and substantiate the complex mechanism of NPM-ALK signal transdution.

Together, our results emphasize the relevance of the identified phosphorylation sites and reinforce the hypothesis that NPM-ALK phosphorylates NIPA to ensure its cytoplasmic localization thereby promoting lymphomagenesis.

## 4. Materials and Methods

### 4.1. Plasmids

Details of the construction of various plasmids are available from the authors upon request. The pCDNA3.1/Zeo Flag hNIPA wild type was kindly provided by T. Ouyang. pCDNA3.1/Zeo Flag hNIPA 1-402 and pCDNA3.1/Zeo Flag hNIPA ∆310–402 were kindly provided by F. Bassermann and A.L. Illert. Point mutations of NIPA were generated by site-directed PCR mutagenesis. pMSCV PIC was kindly provided by C.Miething. MSCV Mig NPM-ALK was kindly provided by J. Duyster.

### 4.2. Cell Culture

Primary NIPA-deficient MEFs and Hek293T cells were cultivated in DMEM containing 10% FCS. Ba/F3 cells were cultivated in RPMI 1640 supplemented with 10% FCS and 2 ng/mL IL-3. Jurkat, FePD, Mac-1, DEL, TS, SUDHL-1, Karpas and JB6 cells were cultivated in RPMI 1640 medium containing 10% FCS.

Transient transfections of primary NIPA-deficient MEFs and Hek293T were performed using TurboFect (Thermo Fisher, Waltham, MA, USA) or Lipofectamine 2000 (Invitrogen, Carlsbad, CA, USA) according to the manufacturer’s instructions.

Generation of retrovirus and transfection was performed as described previously using Turbofect [29]. Ba/F3 cells were stably transduced with retroviral supernatant supplemented with 4 g/mL polybrene (Sigma-Aldrich, St. Louis, MO, USA) for 3 times every 12 h.

Cell synchronization at G2/M was performed by sequential culture with 2 mM thymidine and 40 ng/mL nocodazole and subsequent collection of rounded cells.

Inhibition of PLK1 was performed with Volasertib (Selleckchem, Houston, TX, USA) for 6 h with a concentration range from 0.1 to 10 µM.

### 4.3. Consensus Sequence Analysis

Consensus sequence analysis was performed using the PhosphoNET Kinase Predictor online tool (Kinexus 2017, Vancouver, British Columbia, Canada) for each individual phosphosite.

### 4.4. Flow Cytometry Analysis

For cell cycle analysis, cells were washed with PBS, fixed with 70% EtOH for 12 h at −20 °C, washed again with PBS and stained with 25 mg/mL propidium iodide supplemented with 100 µg/mL RNase. Cell cycle distribution was determined by flow cytometry on an LSRFortessa Flow Cytometer and subsequent analysis using the FlowJo software (Tree Star Inc., Ashland, OR, USA).

### 4.5. Immunofluorescence Microscopy

NIPA (Sigma-Aldrich, St. Louis, MO, USA, HPA024023) antibody was used for immunofluorescence experiments. Alexa Fluor-488 conjugated anti-rabbit IgG was purchased from Life Technologies. Different ALCL cells were fixed on slides with paraformaldehyde and permeabilized with methanol. Cells were stained with primary antibodies for 2 h prior to incubation with fluorophore-conjugated secondary antibodies for 1 h at room temperature in the dark. Mounting of slides was performed using the Gold Antifade reagent (Invitrogen). Slides were viewed with a Zeiss LSM 880 microscope (63×/1.4 Plan-Apo oil objective). Images were processed with Zeiss Zen Black 2.1 software.

### 4.6. Immunoprecipitation and Immunoblot

Immunoprecipitation and immunoblotting were performed as described previously [12,30,31]. Antibodies against β-Actin (A5316) and NIPA (ZC3HC1, HPA024023) were purchased from Sigma. Cul1 (cs-4995), PLK1 (cs-4513) and ALK (cs-3333) were from Cell Signaling. GAPDH (OSG-00033G) was from Osenses, FLAG (PA1-984B) was from Thermo Fisher Scientific. Anti-FLAG-M2 affinity gel was from Millipore. Quantification of immunoblots was performed using LabImage 1D L340 software (Intas Science Imaging, Goettingen, Germany).

### 4.7. MTS Assay and Soft-Agar Assay

Retrovirally infected Ba/F3 cells expressing NPM-ALK and NIPA wt or NIPA S338/344/370/381/T387A were plated on 96-well plates in triplicates (5000 cells in 100 µL RPMI). To assess cell proliferation, MTS reagent (Promega, Madison, WI, USA) was added to the cells after 0 h, 24 h and 48 h and incubated for 2 h at 37 °C. Extinction at 492 nm was measured using a microplate reader (Tecan, Männedorf, Switzerland).

For soft-agar proliferation assays, *Nipa* knockout MEFs were retrovirally infected with vectors containing NPM-ALK and Flag-NIPA wt or Flag-NIPA S338/344/370/381/T387A. The assay was performed as previously described [32]. Between 10^4^ to 10^5^ cells were plated in soft-agar in 6-well plates. Colonies were counted between days 15 and 20 after plating.

### 4.8. Proteomic Analysis

Phoenix cells were transfected with pCDNA3.1/Zeo Flag hNIPA wild type and MSCV Mig NPM-ALK, Flag was immunoprecipitated using Anti-FLAG-M2 affinity gel (Millipore). For the identification of phosphorylation sites of NIPA, samples were prepared with 1 mM DTT for 5 min at 95 °C and alkylated using 5.5 mM iodacetamide for 30 min at 25 °C. Protein mixtures were separated by SDS-PAGE (4–12% Bis-Tris mini gradient gel) and the 55 kDa region of the gel lanes were cut into 2 equal slices. Gel fractions were in-gel digested using trypsin (Promega, Mannheim, Germany) [33]. Digests were performed overnight at 37 °C in 0.05 M NH_4_HCO_3_ (pH 7.5). About 0.1 µg of protease was used for each gel band. Peptides were extracted from the gel slices with ethanol and resulting peptide mixtures were processed on STAGE tips as described [34]. Sample analysis, data acquisition and processing were performed as previously described [35].

### 4.9. Statistics

Statistical comparisons were performed using GraphPad Prism6 software. Data are represented as mean ± SEM. * *p* < 0.05, ** *p* < 0.01, and *** *p* < 0.001, calculated by an unpaired, 2-tailed Student’s *t* test.

## Supplementary Materials

Supplementary materials can be found at https://www.mdpi.com/1422-0067/20/16/4060/s1.
