# Supplementary material for: Proteomic Phosphosite Analysis Identified Crucial NPM-ALK-Mediated NIPA Serine and Threonine Residues
Source: Int J Mol Sci. 2019 Aug 20;20(16):4060. doi: 10.3390/ijms20164060 (PMC6721280; doi:10.3390/ijms20164060)
Supplement: Supplementary file 1 [file ijms-20-04060-s001.pdf]

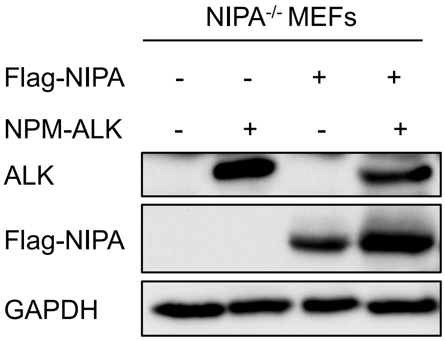

Supplemental Figure 1: NIPA phosphorylation status upon NPM-ALK expression in immortalized MEFs. Immunoblot analysis of NIPA$^{-/-}$ MEFs displaying NIPA phosphorylation indicated by a slight mobility shift in the presence of NPM-ALK.

(A)

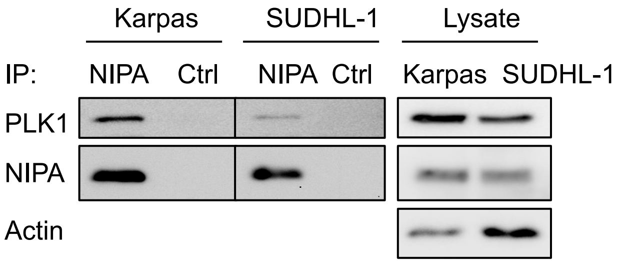

(B)

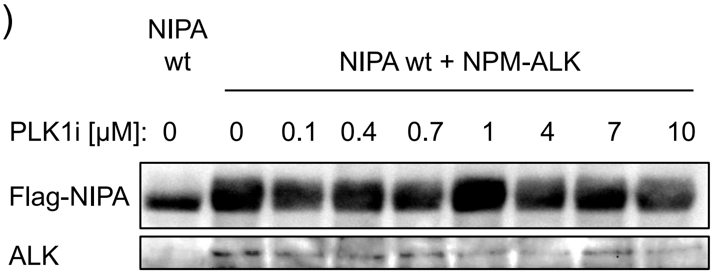

(C)

**Phosphosite**	**Predicted kinases**
S338	ERK2, JNK1, p38MAPK
S344	ERK2, JNK1, ERK1, p38MAPK
S370	p38MAPK, ERK1, JNK1
S381	Pim1/2/3, mTOR
T387	p38MAPK, JNK1, ERK1/2

Supplemental Figure 2: NIPA phosphorylation caused by NPM-ALK expression is mediated by MAP kinases. (**A**) NIPA-immunoprecipitation in Karpas and SUDHL-1 cells demonstrates interaction between NIPA and PLK1. (**B**) Hek293T cells were transfected with vectors containing NPM-ALK and Flag-NIPA followed by treatment with the PLK1 inhibitor Volasertib with indicated concentrations for 6 hours. (**C**) Kinases predicted by consensus sequence analysis of the identified phosphosites by PhosphoNET Kinase Predictor (Kinexus).

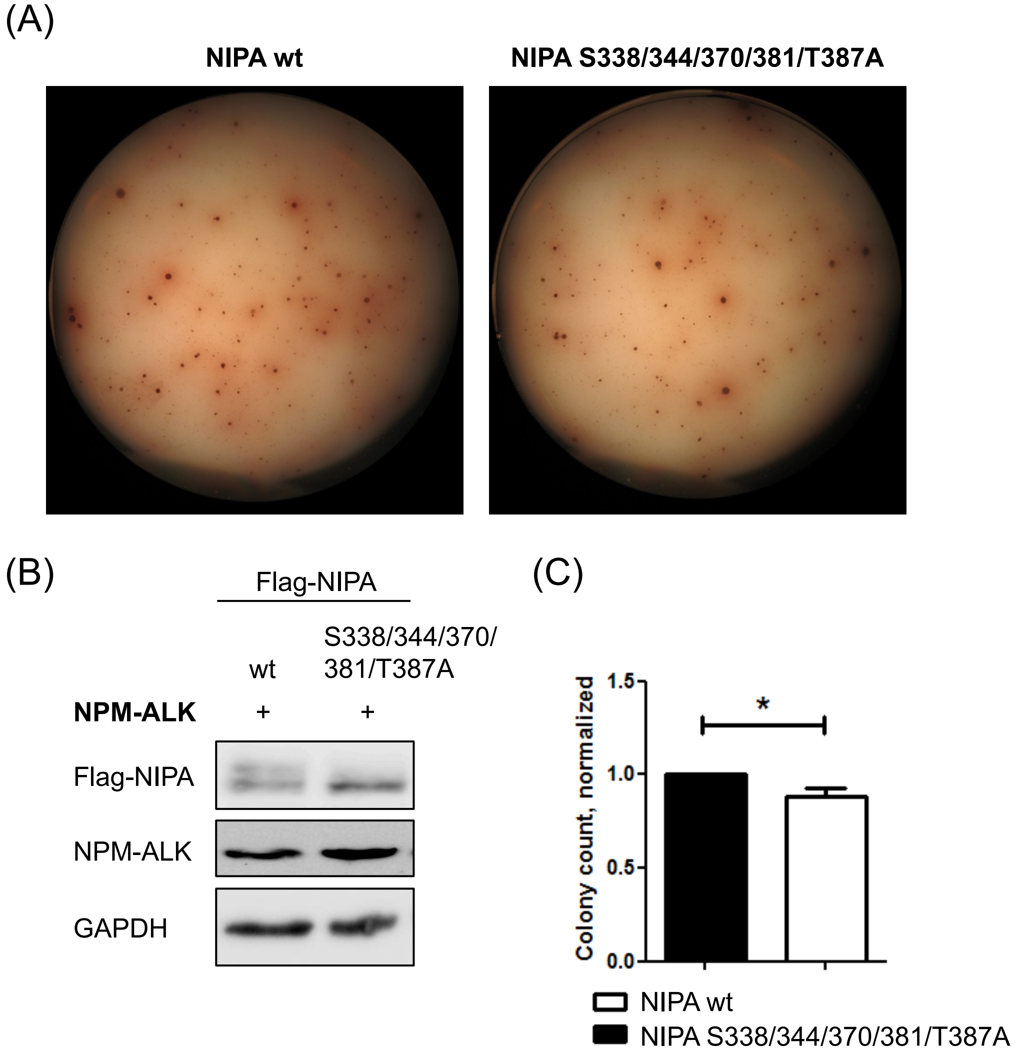

Supplemental Figure 3: NPM-ALK-induced NIPA phosphorylation slightly increases proliferation in NPM-ALK-positive cells. (**A**) Representative images of softagar assays with *Nipa*-/- MEFs expressing NPM-ALK and Flag-NIPA wt or Flag-NIPA S338/344/370/381/T387A displaying elevated colony formation in NIPA wt cells. (**B**) Immunoblot of NIPA-/- MEFs expressing NPM-ALK and NIPA wt or NIPA S338/344/370/381/T387A used for Softagar assays showing equal Flag-NIPA and NPM-ALK expression. (**C**) Quantification of colony formation of duplicates in three independent experiments.
